# Skeletal muscle dysfunction with advancing age

**DOI:** 10.1042/CS20231197

**Published:** 2024-07-12

**Authors:** Pardeep Pabla, Eleanor J. Jones, Mathew Piasecki, Bethan E. Phillips

**Affiliations:** 1 Centre of Metabolism, Ageing and Physiology (COMAP), School of Medicine, University of Nottingham, Royal Derby Hospital, Derby, DE22 3DT, U.K.; 2 MRC-Versus Arthritis Centre for Musculoskeletal Ageing Research (CMAR), U.K.; 3 NIHR Nottingham Biomedical Research Centre (BRC), U.K.

**Keywords:** activity, aging, neuromuscular, physical function, skeletal muscle

## Abstract

As a result of advances in medical treatments and associated policy over the last century, life expectancy has risen substantially and continues to increase globally. However, the disconnect between lifespan and ‘health span’ (the length of time spent in a healthy, disease-free state) has also increased, with skeletal muscle being a substantial contributor to this. Biological ageing is accompanied by declines in both skeletal muscle mass and function, termed sarcopenia. The mechanisms underpinning sarcopenia are multifactorial and are known to include marked alterations in muscle protein turnover and adaptations to the neural input to muscle. However, to date, the relative contribution of each factor remains largely unexplored. Specifically, muscle protein synthetic responses to key anabolic stimuli are blunted with advancing age, whilst alterations to neural components, spanning from the motor cortex and motoneuron excitability to the neuromuscular junction, may explain the greater magnitude of function losses when compared with mass. The consequences of these losses can be devastating for individuals, their support networks, and healthcare services; with clear detrimental impacts on both clinical (e.g., mortality, frailty, and post-treatment complications) and societal (e.g., independence maintenance) outcomes. Whether declines in muscle quantity and quality are an inevitable component of ageing remains to be completely understood. Nevertheless, strategies to mitigate these declines are of vital importance to improve the health span of older adults. This review aims to provide an overview of the declines in skeletal muscle mass and function with advancing age, describes the wide-ranging implications of these declines, and finally suggests strategies to mitigate them, including the merits of emerging pharmaceutical agents.

## Human ageing

As a result of advances in medical treatments and associated policy over the last century, life expectancy has risen substantially and continues to increase globally. In the United Kingdom, one in four adults are predicted to be aged over the age of 65 by the year 2050, and ∼25% of babies born in the last 5 years are expected to reach their 100th birthday [1]. However, the disconnect between lifespan and ‘health span’ (the length of time spent in a healthy, disease-free state) has also increased [2], with losses of skeletal muscle (SKM) mass and function (sarcopenia [ICD-10-CM code: M62.84] [3]) a substantial contributor to this.

Whilst a precise consensus for the definition of sarcopenia, including the exact assessment criteria and standardised cut-offs, has yet to be achieved (reviewed extensively by [4]), it is unequivocal that low SKM muscle mass and function is associated with numerous adverse health related outcomes [5]. These detrimental outcomes impact not only the individuals in question (i.e., via their impacts on mortality, morbidity and independence maintenance/quality of life [QoL]) but also their support networks and at a societal level (i.e., healthcare systems) [6].

## Muscle mass and ageing

### The trajectory of muscle mass loss with advancing age

Age is negatively correlated with both absolute and relative (to whole body mass) skeletal muscle (SKM) mass in both men and women [7,8]. More specifically, cross-sectional data demonstrates that SKM mass (assessed by MRI) remains largely stable until the 5th decade of life, after which it noticeably declines at a steady rate [8]. Although this point of decline is commonly reported across the literature and appears to be largely accepted, it is not unambiguous. For example, some groups report (based on total-body potassium [TBK] and dual-energy X-ray absorptiometry [DXA], or DXA alone) that declines in SKM mass begin as early as the third decade of life [7,9]. Conversely, others report that SKM mass remains largely stable until the age of 60 but declines at an accelerated rate thereafter [10]. The diversity in techniques employed to assess SKM mass and the inherent limitations of cross-sectional studies (i.e., trying to match subjects for confounding factors over a large age-range and across generations) likely explain these discrepant findings. However, largely irrespective of the point of decline, the negative relationship between age and SKM mass is clear [11,12]. It should, however, be recognised that there is substantial variation in this relationship between individuals [13].

In addition to a lack of uniform agreement as to when age-associated declines in SKM mass begin, challenges conducting longitudinal studies make it difficult to determine the precise rates of SKM mass losses. When data from longitudinal studies was assessed (where the average follow-up period across five studies was ∼8 years) the median % decline per year was reported as ∼0.5% in men and ∼0.4% in women across the lifespan. However, this appeared to accelerate to >1% per year after the seventh decade of life [13]. A recent 6-year follow-up study in a German cohort found similar annual rates of decline in older cohorts based on cross-cohort (age-groups) differences in total appendicular lean mass expressed as a percentage of body mass index (BMI), offering support to these earlier reported values [12].

Of note, many studies reporting cross-sectional differences or longitudinal declines in SKM mass with age did not control for physical activity levels and this is likely to be at least partially responsible for the inter-individual heterogeneity observed in these studies. As further outlined in the ‘Mitigation’ section later in this review, it is well documented that levels of physical activity decline with advancing age [14] and that many features of ageing muscle also appear to be features associated with physical inactivity [15]. For example, reductions SKM mass and aberrations in fuel metabolism (i.e., reduced muscle insulin sensitivity [16]) often observed with ageing can be induced (and rapidly) via multiple models of physical inactivity (e.g., step-reduction, limb immobilisation and bed rest) [17]. To illustrate this, following just 7 days of unilateral leg immobilisation via knee brace, a mean reduction in quadriceps volume of ∼7% was observed [18], with a ∼15% reduction in vastus lateralis CSA reported after 15 days [19]. In addition, after just 3 days bed-rest a 17% reduction in whole-body insulin stimulated glucose disposal was reported [20]. Each of these studies, although conducted in young individuals, serve to exemplify the importance of considering physical activity profiles in studies of ageing SKM.

Beyond physical in/activity, there are a number of non-modifiable factors that influence SKM mass and its maintenance with advancing age, with sex one clear example. Although SKM mass is greater in men than women across the life course [8], age-associated SKM mass losses (both absolute and relative) may be greater in men [10,21]. Yet conversely, older females typically have higher rates of frailty and reduced function when compared with men [22–25]. Further, although sex is known to influence regional body composition (i.e., greater gynoid subcutaneous adipose in females), it has been shown that in both men and women, SKM mass of the lower extremities declines at greater rate than in the upper body [8]. There is also evidence to suggest that ethnicity may also impact SKM mass and its maintenance with advancing age. Comparing African American, white, and Hispanic individuals, African American men and women have greater absolute and relative SKM mass compared with their white and Hispanic counterparts across the lifespan (18–80 years). However, it is African American women and Hispanic men that exhibit the greatest age-related declines in SKM mass [9]. In addition, studies of older aged (>65 years), community-dwelling Asian populations demonstrate that they have less SKM mass than their white and black counterparts, but that age-related declines in SKM mass are broadly similar across these ethnic groups (0.2–1% per year) [26]. Beyond ethnicity, there is evidence to suggest that SKM mass is a phenotype with high heritability (estimates of 52–60%) [27,28]. However, there are relatively few studies exploring the genes associated with SKM ageing [29], and of those that do, the focus is often on combined bone and muscle outcomes [30,31], rarely SKM maintenance and very few utilising up-to-date sarcopenia definitions [32].

### Clinical and societal implication of reduced muscle mass

The largely inevitable (to a degree [33]) loss of SKM mass with advancing age can have major implications at both the clinical (i.e., for the individual) and societal level. Indeed, SKM mass, independent of fat mass and cardiovascular and metabolic risk factors has been shown to be inversely associated with all-cause mortality in older adults [34]. Similarly, a recent systematic review and meta-analysis of 16 studies conducted across the world (including countries in Asia, North America, South America, Europe, and Australia) concluded that low skeletal mass index (SMI; skeletal muscle mass per unit of height) was associated with increased all-cause mortality, even when stratified by age categories (i.e., in people aged less than 45 years, aged 45–65 years, and in those over the age of 65 years). The pooled relative risk of all-cause mortality was 1.57 (95% CI: 1.25–1.96) in those with the lowest SMI and the risk of all-cause mortality associated with low SMI was greatest in those with a higher BMI [35]. Subgroup analysis of those over the age of 65 years demonstrated that a low SMI could increase all-cause mortality risk by 56%, clearly illustrating the importance of SKM mass for longevity. Given the relationship, albeit not linear, between muscle mass and function losses, it is perhaps not surprising that in addition to low SKM mass being predictive of negative health outcomes, especially in older adults, the same is also true for muscle function [36,37]. Although studies regarding longevity do support the importance of SKM mass maintenance for older adults, enhanced health-span is arguably a more important target than lifespan. Current data suggests that older adults spend the last ∼9 years of their life with illness and disability [6], with clear inequalities associated with wealth and ethnicity [38,39]. It is, however, clear that SKM mass is associated with health, not just lifespan [40].

The importance of SKM mass maintenance with advancing age is perhaps even more important for older patient cohorts (i.e., compared with community-dwelling individuals). For example, the iLSIRENTE study from Italy showed that in community-dwelling octogenarians muscle mass was predictive of survival over a 7-year follow up period [41]. Similarly, a study by Gakhar et al. showed that in older adults with spinal cancer, muscle mass was predictive of survival one year after surgery for the cancer even when the data were corrected for tumour and nodal scoring [42]. Further, a large-scale observational study from Geneva looking at both medical and surgical admissions showed that low SKM mass was predictive of both hospitalisation in the first place and adverse clinical outcomes (e.g., mortality) [43], a finding recently echoed by other groups in difference clinical cohorts [5,44]. Ageing is a major risk factor for numerous conditions (i.e., falls and non-communicable diseases) requiring hospitalization [45,46]. Hospital stays, even without an acute catabolic insult such as infection or surgery [47], are associated with SKM mass losses, likely due to them representing a prolonged period of inactivity (often in the form of bed-rest). Further, when this activity is combined with significant systemic illness, such as that seen in the critical/intensive care setting these losses of SKM mass are greater still [48]. It is likely that in many patients, dietary inadequacies during hospitalisation also contribute to the rapidity of observed SKM mass losses. With contractile activity and protein nutrition the two most potent anabolic drivers [49,50], just 3 days of muscle disuse have been shown to elicit significant SKM mass losses [18]. With inactivity (or SKM disuse) a common experience in hospitalised patients, when this is combined with inadequate nutrition the resultant SKM atrophy is both significant and clinically relevant [44]. A recent observational study by Hardy et al., in an older post-surgical (for colorectal cancer resection) cohort reported in-hospital nutritional intake in the 5 days after surgery as only ∼25% of ESPEN guidelines for energy and only ∼12% for protein [51]. Comparing young with older adults, it has been shown that in response to SKM disuse older adults lose SKM mass at a markedly faster rate (∼3–6 times) [52]. In addition, older adults are not able to fully recover losses of skeletal muscle at the same rate or as completely [53]. As such, a combination of ‘normal’ age-associated SKM mass losses paired with periods of hospitalisation may accelerate declines in SKM mass taking individuals across a disability/dependency threshold prematurely [54].

### The mechanistic basis of muscle mass loss with advancing age

SKM mass is maintained by the dynamic equilibrium that exists between rates of muscle protein synthesis (MPS) and muscle protein breakdown (MPB) [55]. Therefore, any imbalance in these two processes can impact net muscle protein balance. When MPS exceeds MPB this leads to muscle protein accretion and subsequent hypertrophy. Conversely, when MPB exceeds MPS resultant muscle atrophy will occur. As briefly alluded to in the section above, the two key ‘environmental’ stimuli for stimulation of MPS are: (i) the ingestion of dietary protein and (ii) muscle contractile activity (e.g., exercise or physical activity), with the greatest stimulatory effect coming from a combination of the two [56].

Although both MPS and MPB contribute to the dynamic equilibrium needed to maintain SKM, it appears as though age-associated declines in SKM mass are primarily driven by alterations in MPS, with the same appearing to be true for inactivity-induced atrophy [57]. More specifically, it appears that although ‘basal’ (rested, postabsorptive) rates of MPS do not differ between young and older individuals [58], the muscle protein synthetic response to both protein feeding [49,59] and resistance exercise (RET; the most potent form of contractile activity to stimulate MPS [50]) is blunted in older age. This ‘anabolic blunting’ has been shown in both men and women [59,60], with some suggestions that higher basal rates of MPS explain the relative protection of SKM mass in older women (versus men) [61]. This attenuation in MPS responses to anabolic stimuli is also accompanied by reduced phosphorylation of key signal transduction proteins in the mammalian target of rapamycin (mTOR) signalling pathway [49]; a key signalling pathway in muscle anabolism ultimately leading to greater amino acid translation into structural protein [62]. However, it should be acknowledged that a dissociation between anabolic signalling and muscle protein metabolism has been reported [63,64].

One methodological consideration deserved of attention is that each of the aforementioned studies reporting MPS employed acute stable isotope amino acid tracers to assess this, and as such only provided measures over a relatively low number of hours (i.e., <24 h, typically 2–8 h) [65]. However, in recent years, the development of pyrolysis–isotope ratio mass spectrometry for the high-precision measurement of hydrogen and oxygen stable isotopes has led to the re-introduction of the first stable isotope used in metabolic research, deuterium oxide (D_2_O) [66]. Compared with the ‘traditional’ amino acid tracers which require continuous intravenous infusions that prohibit their use over prolonged free-living situations, D_2_O is orally ingested and can facilitate measurements over many days and weeks [67,68]. In relation to age associated SKM mass losses, not only has D_2_O been used to show anabolic resistance to RET in older age [69] but is also a key part of the recently developed COSIAM (Combined Oral Stable Isotope Assessment of Muscle) methodology which can simultaneously determine whole body muscle mass, MPS and MPB, including in older adults [70,71]. Specifically, Brook et al. showed that after 6-week RET, older individuals had reduced rates of cumulative MPS, attenuated translational efficiency and capacity, as well as lower levels of anabolic hormones; all of which underpinned blunted SKM hypertrophy compared with that seen in younger individuals [69]. As illustrated by this work, the pathophysiology of age associated SKM mass losses is undoubtedly complex, with emerging concepts adding to this ‘puzzle’. For example, Abdulla et al. showed that glucagon like peptide-1 (GLP-1) infusions can overcome anabolic resistance to feeding in older men, with the postprandial insulin, hyperinsulinaemic clamp conditions employed in this study leading to a suggestion that GLP-1 may have a direct effect on muscle at the cellular level [72].

Beyond SKM mass losses, another body composition change that occurs with advancing age is an increase in fat mass and in particular, lipid infiltration of SKM muscle tissue [73]. This infiltration (myosteatosis) has been shown to be associated with impaired muscle contractile function [74] and the development of insulin resistance [75], which is associated with attenuated MPS responses to nutrition [76]. Further, obese older men have been shown to exhibit lower postprandial MPS under hyperinsulinemic hyperaminoacidemic–euglycemic clamp conditions versus healthy weight counterparts. However, lower rates of MPB were also observed in the obese men, such that net protein balance was not different between the groups [77]. Whether the attenuation of MPS with elevated lipid availability is mediated via a direct impact of lipid accumulation on the mechanisms underpinning increased MPS, or if the lipids modulate insulin sensitivity remains to be elucidated. It should however be noted that neither acute (i.e., via the oral ingestion of a lipid emulsion) nor short-term (2 weeks) fat over-feeding is associated with impaired MPS responses to protein in overweight/obese men [78]. Therefore, it appears that dietary-induced accumulation of intramuscular lipids per se (i.e., without the manifestation of insulin resistance) is not associated with anabolic resistance. Furthermore, in overweight individuals, anabolic resistance to protein ingestion induced through acutely raising lipid availability via a 5 h intravenous lipid infusion of can be overcome by a single bout of (combined cycling and resistance) exercise in overweight middle-aged men [79]. This serves to highlight the potential role that maintained physical activity may play in mitigating any detrimental effects of excess lipid availability on MPS.

One area that has been intensely studied in recent years is to what extent the declines in muscle mass and metabolic functions that occur with advancing age are attributable to the reduced physical activity also seen in older adults [14]. Inducing inactivity through, for example, limb casting leads to rapid (within days) blunting of MPS and loss of muscle mass in healthy young individuals [18,80], clearly exemplifying the need for contractile activity to achieve muscle mass maintenance. Although limb casting may be considered an extreme model of disuse, even just reduction in step count, a much milder form of inactivity, also induces anabolic resistance and significantly reduces leg fat free mass. Furthermore, reduced step count also impairs insulin sensitivity and heightens systemic inflammatory profiles, both of which may contribute to detrimental muscle metabolism, with these data acquired in a study of community-dwelling older individuals (∼76% step-reduction from habitual activity) [81]. As with age-associated losses of SKM mass, the precise mechanisms by which inactivity leads to muscle atrophy are yet to be determined, with this endeavour further complicated by the phenomena of atrophy resistant versus atrophy susceptible (aRaS) muscles [82]. There is, however, a growing evidence base to suggest that neither basal nor postprandial rates of MPB have a significant role to play in disuse-induced muscle atrophy [83,84]. Therefore, strategies to combat this should focus on the augmentation of MPS rather than the attenuation of MPB.

## Muscle function and ageing

Clearly, when considering SKM declines with advancing age, the maintenance of SKM function is as important, if not more so, than the maintenance of SKM mass. In support of this, the most recent definition of sarcopenia from the European Working Group on Sarcopenia in Older People (EWGSOP2), updated (from 2010) based on research evidence to: ‘a muscle disease (muscle failure) rooted in adverse muscle changes that accrue across a lifetime’ [85,86], and placed muscle function at the forefront of the criteria for sarcopenia diagnosis (compared with previous definitions placing greater focus on muscle mass [87]). That diagnosis of the condition now focuses on low muscle strength as a key characteristic which reflects the observation that muscle strength decreases at a substantially faster rate than size [13,85], highlighting the complex interplay of different features (beyond muscle size) that contribute to optimal muscle function.

### Assessing muscle function with advancing age

The term SKM function (without any predication, i.e., SKM metabolic function) is most commonly used to describe SKM muscle contractile properties and/or the resulting actions. For example, the assessment of muscle strength is commonly employed as a measure of muscle function in research exploring the impact of ageing and/or interventions [11,36]. However, even for just this single measure (the production of force), a number of different assessments can be conducted (as is true for SKM mass [88]), rendering comparisons between different studies somewhat difficult. For instance, based on their clear importance in ambulation and activities of daily living (e.g., rising from a chair), the knee extensor muscles are often the focus of strength assessments in older adults [89]. However, even within this single muscle group, strength measures can still vary, with strength (or peak torque) able to be assessed (i) at a fixed angular velocity which allows a dynamic measure of lower limb function; (ii) isometrically by measuring the maximum force that can be applied against an immovable object; or (iii) with no fixed velocity in the form of 1-, 5- or 10-repetition maximum assessment (i.e., 1-RM). Further, strength is far from the only component of muscle function, with power (the product of force and velocity), balance, flexibility, stamina, agility, and dexterity/force accuracy all associated with health-related outcomes in older adults [90,91]. In addition, and as discussed in more detail in sections below, adaptation of the neural input to muscle must be considered as a potential factor to explain the frequently reported disparity in muscle mass and functional losses seen with advancing age [21,92].

Beyond the challenge of drawing conclusions based on heterogeneous assessments of muscle function, there are also specific challenges when assessing physical function in older individuals, especially those with frailty and or joint/mobility limitations. Using knee extensor strength as an example, this is most commonly assessed using some form of seated leg extension. However, for individuals with joint limitations at the hip or knee, simply positioning themselves to be able to complete this assessment may be challenging, and the knee extension action may cause discomfort and potentially harm [93]. Considering that up to 25% of individuals over the age of 55 have hip osteoarthritis (OA) and a similar number report persistent knee pain [94], this is a noteworthy consideration given its inhibitory effects on muscle contraction [95]. These limitations can even exist for assessments that outwardly appear relatively innocuous. Handgrip strength for example is often considered a safe and easy-to-perform assessment of physical function, especially when compared with those using larger muscles groups and/or specialist equipment. Shown to correlate with mortality, fracture risk and hospital length of stay, handgrip strength is often reported in large scale studies of older adults [96] where perhaps the resource/logistical burden of other measures of SKM function would not be possible. However, for those with OA of the hand (∼20% of those over 70 years [97]), this may prove very difficult. There are however a number of tools which have been designed and/or validated for the specific purpose of evaluating physical function in older adults. The most used of these include the 6-min walk test (6MWT) [98], timed up-and-go (TUG) [99], and the short physical performance battery (SPPB) which includes measures relating to walking speed, balance and ability to rise from a chair unaided [100]. Whilst these measures have each shown good agreement with lower limb strength and do not stress joints to the same extent as strength assessments for example, a ‘ceiling effect’ may be encountered if these methods are used in high-functioning (i.e., independent community-dwelling) older adults [101] who commonly participate in research studies.

### Declines in muscle function with advancing age

In contrast with the more subtle (but certainly not trivial) declines in SKM mass in the latter decades of life, indices of muscle function (most notably strength and power) decline at a much faster rate and as such may have greater detrimental consequences for older adults [12,102]. This dissociation between the magnitude of SKM mass and functional losses across the lifespan has now been illustrated in a relative wealth of studies, with cited values of ∼1% /year for SKM mass losses beyond the age of 70 compared with >3% for strength [13,102,103]. For example, Goodpaster and colleagues showed that over a 3-year period, the annual loss of isokinetic knee strength was ∼3 times greater than the 1% loss of leg lean mass in both men and women [21]. Further, as the rate of strength loss accelerates across progressive decades of later life so does the difference in loss of strength relative to loss of SKM mass [104]. A 5-year follow-up of over 300 Korean men and women over the age of 65 showed that losses of leg strength (isokinetic knee extensor strength at an angular velocity of 60°/s) accelerated dramatically after the age of 70 in men and 75 in women, where it reached ∼5% per year at the age of 80 and greater than 10% per year at 90 [102]. Beyond SKM strength per se (which is associated with a multitude of health-related outcomes) strength loss has been shown to predict fracture risk in elderly individuals [105], which in turn is associated with increased disability and mortality [106].

Considering SKM power, recent evidence suggests that declines in this component of SKM function are not only greater that those seen for strength but also begin earlier in adulthood. Based on longitudinal assessments of strength (handgrip strength) and power (two-legged jumps and chair-rise force) in over 300 men and women over a 6-year follow-up period, Wiegmann and colleagues found that in the oldest age-group (>70 years), power declines (male data: 2.76% for the chair rise test and 3.34 for the two-legged jump) were approximately double that seen for strength (1.48%). Interestingly, although this disparity between strength and power loss was similar in females of the same age, changes in absolute strength were significant in women by the age of 50, yet this was not seen until the age of 70 in men. In contrast, declines in muscle power could be seen in the youngest cohort (20–39 years) for both sexes [12]. Confirming these early losses of muscle power, a recent 10-year longitudinal study demonstrated declines in maximum power of the knee extensors in young (20–39 years), middle-aged (40–59 years), and older (60+ years) individuals [107]. In the young group, this loss of power was accompanied by reduced force production only, whereas in the older age groups it was associated with declines in both force and velocity.

### Clinical and societal implication of declines in muscle function

Given the global trend of an ageing population and the undeniable disconnect between lifespan and health-span enhancement as detailed in the opening section of this review, maintenance of skeletal muscle function needs to be afforded significant attention given its vital role in mobility, independence, quality of life, metabolic functions [108] and seemingly even cognitive health, for which ageing is a major risk factor. Indeed, in a 10-year follow-up study of female twins, baseline leg power was associated with improved cognitive ageing [109] independent of SKM mass [110].

As is seen for the relationship between SKM mass and all-cause mortality and disability [36,37], a robust relationship between muscle function and these detrimental outcomes also exists [111]. Indeed, as is seen for SKM mass in older community-dwelling [41] and patient cohorts [42], loss of SKM muscle function is associated with an elevated risk of hospitalisation and mortality, often based on the development of frailty [2]. For older individuals, the ability to perform everyday tasks and maintain independence is often their ageing priority [112], with clear socio-economic impacts of prolonged independence. For example, in the UK alone, the cost of care home residency for older adults is estimated at £972 per week (based on an internal report by Lottie.org [113]). Difficulties in performing basic activities of daily living not only increase the need for personal assistance or relocation to a supportive (e.g., care home) environment [114–116] but also have numerous adverse effects on health status and mortality [117]. Specifically, analysis of longitudinal data from a database of epidemiological studies in older adults showed that inability to independently perform activities of daily living results in diminished survival and more of that survival period spent in disabled states [118].

Yet again replicating what is seen with SKM mass losses, periods of inactivity, which in older adults often occur due to hospitalisation and/or bed-rest, further exacerbate declines in SKM function. Healthy older individuals subject to 10 days of bed-rest experienced a 13% reduction in lower extremity (knee extensor) strength and had reduced (14%) stair climbing power [52]; with these rates of loss greater than that reported in younger individuals following bed-rest [119]. In addition, older adults also exhibited decreased voluntary physical activity levels after bed-rest [52], hindering potential recovery of SKM function. In relation to functional recovery, it has been demonstrated that compared to younger individuals, older (surgical) patients regain muscle function more slowly and less completely [120]. Therefore, older patients who likely have lower muscle function by virtue of age, experience accelerated losses due to disuse scenarios and also have to contend with incomplete recovery. This combination conceivably predisposes older adults to development of sarcopenia, frailty, loss of independence and overall, a reduced quality of life- all of which beget substantial burden for individuals, families, and healthcare systems/society [121,122]. Exemplifying the economic burden of SKM functional losses, in the UK alone the estimated annual excess burden associated with muscle weakness is ∼£2.5 billion [123].

### The mechanistic basis of declines in muscle function with advancing age

It is clear that losses of SKM function have detrimental impacts at many levels, however as with losses of SKM mass, the precise contributing mechanisms are not yet fully understood. As already mentioned, there is a clear disparity between rates of SKM mass and function losses seen with advancing age. This observation highlights that loss of mass cannot be the sole determining factor for losses of muscle function, with changes in muscle structure/quality (outside the scope of this review) and adaptation of the nervous system likely having a significant role to play [124].

### Declines in neuromuscular function with advancing age

Neurological changes with advancing age can impact the motor cortex and descending tracts, spinal motor circuitry and motoneuron activation, and the motor unit (MU); with the term MU encompassing both the motoneuron and the muscle fibres it innervates [125]. Studies using intramuscular electromyography (iEMG) to obtain information from a range of different MUs at different muscle depths [126,127] or near fibre recordings [128–131] for estimations of size, number, and structural and functional characteristics, have established notable differences in older MUs, when compared to young [132].

#### Central motor control and advancing age

Excitatory output signals from the primary motor cortex ultimately lead to muscle contraction following transmission down descending tracts, synapsing at the spinal circuitry, with both excitatory and inhibitory corticospinal inputs influencing synergistic muscle coordination [133]. In this context, ageing is generally characterised by reduced cortical activation and decreased voluntary activation presenting functionally as declines in both muscle strength and motor control [134]. However, central motor control is complex in nature, with numerous adaptations occurring with older age, from brain activity to motoneuron excitation, each of which are highly task dependent [135].

Persistent inward currents (PIC) act to prolong and amplify synaptic inputs to motoneurons [136], and estimates in humans show reduced PICs in both upper [137] and lower [138–140] limbs of older individuals compared to young. This has probable implications on rate coding, the alteration of MU firing rate (MUFR) in response to task demands. Indeed, several independent human studies have demonstrated lower MUFR at normalised contraction levels in old compared with younger individuals (e.g., [138,141].). However, yet again this appears to be task, muscle, and contraction intensity specific [142] limiting the robustness of conclusions surrounding age-associated changes. Larger age-related difference at higher contraction intensities undoubtedly limits maximal force generating capacity and as such, likely explains a currently unknown portion of strength deficits in older muscle.

In addition to assessing maximal strength, evaluating the capacity to sustain target forces at low or submaximal contraction intensities is used to further assess neuromuscular function [143]. This measure of force steadiness/accuracy describes the inherent fluctuations around a constant force output, and again, ageing is generally characterised by poorer performance in these tasks [144]. Definitive mechanisms for this are not fully established, but are postulated to include an increase in MUFR variability (defined as the co-efficient of variation of the inter-spike-interval) and variations in common and independent synaptic inputs to the MU pool [143].

#### The neuromuscular junction and advancing age

The NMJ is a specialised chemical synapse which transmits signals from motoneurons to postsynaptic nicotinic acetylcholine receptors on the muscle fibre membrane to stimulate contraction. Direct structural imaging of the NMJ in humans is notoriously complex and often requires cadaveric or post-amputation limbs [145], and therefore the majority of age-related data in this field has been generated from animal models. These synapses demonstrate high plasticity and undergo both morphological and physiological degradation in structure and function with advancing age [146–148]. These age-associated changes include declines in mitochondrial number and changes in vesicle number in the presynaptic terminal (decreased) postsynaptic endplate area (increased) [149]. Studies are equivocal in other aspects, with some reporting both larger and more complex post-synaptic regions in older age [150], whilst others show no differences [151,152]. Histochemical analysis of the NMJ in aged animal models has found NMJ fragmentation [153], although the relevance of this in humans and for neuromuscular transmission instability and subsequent muscle activation has been questioned [154].

Aged rats have been shown to exhibit increased NMJ transmission instability and reduced reliability which correlated with declines in functional measures such as grip strength [155]. In humans, functional NMJ transmission instability can be measured *in vivo* via intramuscular EMG (iEMG) using a statistic (‘jiggle’) representative of the variability in consecutive MU [156] or near fibre MU potentials [128]. Although human data related to age-associated changes in NMJ function is still relatively limited (but an area of growing interest), cross-sectional iEMG data highlight increased NMJ instability in the lower limb muscles of older adults [129,130,132], which may reflect early stages of SKM fibre denervation-reinnervation.

#### Motor unit remodelling and advancing age

The number of functioning MUs decreases with advancing age [126], yet they also demonstrate high levels of plasticity and are capable of structural remodelling [157]. Following MU loss, denervated muscle fibres may be reinnervated by adjacent surviving axons leading to enlarged MUs (in terms of MU fibre ratio) [158]. In addition, MU potential complexity also increases with both advancing age and exercise. This indicates the temporal dispersion of propagating muscle fibre action potentials which is a measure representative of MU remodelling [126,127,129]. It is unclear what direct functional effects MU remodelling may have, yet increased NMJ transmission instability and a greater number of muscle fibres within a MU as a direct result of this process may feasibly impair motor control [159,160]. Additionally, MU remodelling is suggested to be responsible for a greater proportion of type 1 fibres in older compared with younger muscle as observed in both animal models [161,162] and human studies [163], although this is not a consistent finding [164]. This change in fibre type proportion may contribute to the reductions in muscle power observed in older age.

## Strategies to mitigate age-associated declines in skeletal muscle

As the proportion of older people in the population increases, the need for interventions to maintain SKM ‘health’ (mass and function) into older age is more pressing than ever [165]. To date, a number of classes of intervention aiming to mitigate age-associated losses of muscle mass and function have been employed and evaluated both in isolation and as combinations. In broad terms, these classes include: (i) contractile strategies (both voluntary and involuntary), (ii) nutritional strategies, and (iii) pharmacological strategies.

### Contractile strategies

Considering voluntary contractile strategies, exercise is a well-accepted and promoted intervention for increasing and/or mitigating losses of both SKM mass and strength with advancing age. As an umbrella term for multiple forms of structured physical activity, each of which elicit distinct physiological adaptation [166], the ‘traditional’ division of exercise type was aerobic exercise training (AET) and resistance exercise training (RET), although it should be noted that high intensity interval training (HIIT) is a format of exercise receiving growing attention in the field of exercise-for-health [167,168].

RET is often considered the ‘gold-standard’ intervention for preservation of muscle health across the life course [169]. However, although RET has been demonstrated to be an effective intervention for improving both muscle strength and promoting neuromuscular plasticity in people of all ages [157,170], hypertrophic responses in older adults are often blunted when compared with younger individuals [60]. For example, Phillips and colleagues reported that despite complete engagement with a fully supervised programme of 20 weeks whole-body RET, only young individuals achieved significant hypertrophy (compared with middle-aged and older adults), despite almost identical (relative) functional gains [171]. SKM hypertrophy is known to be achieved through increases in MPS; however, additional proposed underlying mechanisms for exercise-induced hypertrophy include metabolic stress, mechanical signals, and muscle damage which can stimulate the up-regulation of both anabolic hormones and signalling pathways [172]. However, which of these, if any explain the adaptive blunting seen in older adults is not yet known. However, given the consistent observation that increases in SKM function occur prior to detectable changes in SKM mass this suggests a key role of neural adaptations for functional mal/adaptation [173,174]. Finally, recent evidence suggests that, despite anabolic resistance to RET being a well-reported feature of advancing age in both men [50] and women [175], just four acute sessions of RET in the week prior to a (5 days) period of inactivity is able to somewhat preserve acute MPS responses to nutrition at the end of the inactivity period. This strategy did not, however, increase ‘integrated’ MPS rates across the inactivity period, nor did it preserve SKM mass (quadriceps cross sectional area) [176]. Offering more promise in terms of SKM mass maintenance, more recent work from the same group has shown that just a single session of RET the night before a 5-day period of inactivity can attenuate losses of muscle mass and declines in integrated MPS. However, if this strategy is feasible and effective in clinical situations of disuse, including those where underlying conditions may impact SKM metabolism (e.g., colorectal cancer [177]), and the impact on muscle function, is still to be determined.

Despite being most strongly associated with improvements in cardiorespiratory fitness (CRF) [178,179] and insulin sensitivity [180,181], AET has been demonstrated to preserve both muscle mass and function in older adults. Preservation of SKM function with AET has been suggested to be due to increased oxidative capacity and improved mitochondrial efficiency [182]. In a similar way to AET, HIIT (which is also effective at improving CRF [183] and IS [184]) has also been shown to improve SKM mass and function in older adults through proposed mechanisms of increased MPS and improved mitochondrial oxidative capacity, respectively [185].

Somewhat irrespective of exercise modality, challenges do present in promoting/facilitating exercise uptake and adherence [186], some of which are specific to older adults. For example, although RET, AET and HIIT have each been shown to be feasible and effective in older adults with different levels of ability [187], common age-associated conditions such as, for example, OA or uncontrolled hypertension may limit individuals ability to safely participate [188,189]. Further, beyond physical limitations, many older adults lack confidence and motivation, as well as having restrictions relating to transport and finance [190].

In addition to long-term age associated conditions which may prove challenging for exercise training and/or necessitate adaptations to traditional training regimes (e.g., resistance bands instead of free weights for RET [191,192]), traumatic events such as fractures or surgery (both of which are common in older age) which can result in immobilisation or bed-rest add another layer of complexity to the delivery of exercise training [54]. In these situations, or indeed any situation whereby older adults are not able to perform voluntary contractile activity, involuntary stimulation of muscle contraction may hold promise. Neuromuscular electrical stimulation (NMES) is a passive contractile strategy whereby electrical pulses are applied to the nerve, or more commonly the SKM belly, via skin electrodes causing depolarisation of motor axons and/or muscle fibres that results in contraction [193,194]. NMES has been shown to minimise muscle size and strength losses [195,196] of older adults via suggested mechanisms of IGF-1-induced MPS [197] and increased SKM oxidative enzyme capacity [198]. NMES has been demonstrated to be an effective technique for both improving metabolic [199] and physical function in older clinical populations with limited ability to exercise [200], including those recovering from major abdominal surgery [201].

### Nutritional strategies

Protein is the main component of SKM and as such intake of dietary proteins, including those from both animal and plant sources, is a prerequisite for the maintenance of SKM mass with advancing age [202]. The ability of dietary protein to enhance SKM mass and function is particularly potent particularly when combined with RET [203]. For example, in older adults, protein intake at or above the current recommended daily allowance (RDA) has been shown to be associated with improved SKM mass maintenance and functional performance [204,205]. Further, when RET is combined with protein supplementation gains in both SKM mass and function by older adults have been shown to be superior when compared with RET alone [206]. Indeed, based on a growing body of evidence suggesting that dietary protein needs increase with advancing age, the most recent ESPEN (European Society for Clinical Nutrition and Metabolism) guidelines on clinical nutrition in geriatrics suggest that protein intake should be at least 1 g/kg body weight/day [207]. When considering the components of dietary protein (i.e., amino acids [AA]), it is the essential AAs (EAA) that are needed as they are not synthesised in the body. It is one of these EAA, leucine, that is recognised as the most potent dietary stimulator of MPS [208,209] via stimulation of the mTOR signalling cascade [202], and as such is often suggested as a key nutritional strategy to maintain muscle mass in advancing age. However, despite leucine being able to stimulate MPS in both young and older adults [209,210], without the addition of RET and/or adjuvant dietary supplementation (e.g., vitamin D [211]), the evidence for leucine alone being able to preserve muscle ‘health’ in older age is lacking [212]. Similarly, acute HMB (β-hydroxy-β-methylbutyrate), a metabolite of leucine, has been shown to both stimulate MPS and attenuate MPB [213]. Further, in longer-term supplementation trials, HMB has been shown to improve SKM mass and function across the continuum of clinical care (i.e., in-patients, outpatients and community-dwelling [214–216]) with a focus on older adults. However, it should be noted that much of this evidence is based on HMB being given with adjuvant nutritional support (most commonly a mixed macronutrient oral nutritional supplement) or RET [217].

Another nutritional compound which has been reported to offer benefit for SKM in older age is omega-3 polyunsaturated fatty acids [218]. The benefits of omega-3 polyunsaturated fatty acids on cardiovascular health are well-established [219] and have now been demonstrated to extend to SKM. Indeed, relatively recent studies have shown that they can improve both SKM mass and strength in older people [220,221]. Proposed mechanisms of action for these improvements include the activation of mTOR signalling [222], and the anti-inflammatory properties of omega-3 polyunsaturated fatty acids improving mitochondrial function and increasing nerve conduction velocity [223].

Additional ‘nutraceutical’ compounds have also shown some potential to enhance muscle mass (creatine, vitamin D, ursolic acid, and phosphaditic acid) and function (carnitine, creatine, nitrates, and β-alanine) [224]. However, the evidence for many of these in older adults is limited, and full discussion on each of these is outside the scope of this review.

### Pharmacological strategies

To date, RET with supportive nutrition is the most successful strategy to maintain SKM mass and function with advancing age, with evidence for other options, especially in humans, remaining relatively scarce. There is however growing interest in pharmacological strategies as reviewed by Leuchtmann and Handschin in 2020 [225]. One such strategy that has received such attention for a number of years is the provision of anabolic hormones based on the decline in naturally occurring hormones such as testosterone and growth hormone with advancing age. A number of studies have shown that anabolic hormone supplementation can improve both SKM mass and function in older age [226,227], with these studies conducted in both older men and women. Testosterone in particular is the male sex hormone, and following declining endogenous levels with advancing age, exogenous supplementation in older men has been shown to elicit increases in muscle mass and strength [228,229]. Suggested mechanisms by which testosterone supplementation improves SKM mass and function include reduced expression of ubiquitin ligases which are responsible for MPB [230] and enhanced myogenic gene programming, myocellular translational efficiency and capacity, higher protein turnover, and net protein increase when testosterone as administered adjuvant to contractile activity [231]. Beyond testosterone, human growth hormone and insulin like growth factor 1 (IGF-1) have also each been shown to increase muscle mass in older adults via enhanced MPS responses to nutrition and exercise [232,233].

More recently, the use of pharmaceuticals to try and combat age-associated losses of SKM mass (primarily) and function has focussed on the repurposing of available and licensed drugs [234], including rapamycin. Rapamycin is an allosteric mTOR inhibitor which is routinely used for its potent immunosuppressive properties (i.e., in organ transplant recipients) which is achieved through binding to FKBP12, the 12-kDa FK506-binding protein, which inhibits mTORc1 to prevent mTOR signalling. Although mTOR signalling is commonly recognised as key component in the *promotion* of MPS and subsequent SKM hypertrophy, the enhanced chronic mTORc1 activation seen in older adults may stimulate progressive muscle damage and loss [235], thus offering explanation as to how mTOR inhibition may be beneficial for older adults [236]. In pre-clinical models, rapamycin treatment has been shown to extend lifespan [237], counteract muscle loss [238], and maintain muscle function [239]. Further, rapamycin treatment has also demonstrated positive effects on the NMJ through increasing AChR density and promoting axonal sprouting through gene up-regulation [239,240]. However, further research in humans is required to validate the safety and effectiveness of rapamycin as an intervention to mitigate age-associated losses of SKM mass and function.

Two drugs which are commonly used for the treatment of diabetes have also shown potential in the mitigation of SKM mass and function losses, metformin and semaglutide. Metformin is an anti-hyperglycaemic agent which has also shown anabolic effects on muscle in older adults, particularly those with diabetes, as well as improvements in health and lifespan in mice [241,242]. The mechanism of action is thought to be via increased AMPK signalling and blunted mTORc1 signalling in a similar way to rapamycin which promotes autophagy [243]. However, it may limit hypertrophic responses of SKM muscle to RET as demonstrated by a greater increase in thigh muscle in older adults taking a placebo rather than metformin whilst performing 14 weeks RET [244]. Semaglutide is a glucagon-like peptide-1 (GLP-1) receptor agonist that mimics the GLP-1 hormone which is released in the gut in response to eating. Used as therapy for both Type 2 diabetes and obesity, semaglutide stimulates insulin production to reduce blood glucose, supresses glucagon secretion, and slows gastric emptying which increases satiety. In observations relating to its effectiveness at reducing obesity, semaglutide has been shown to reduce adiposity whilst preserving SKM mass [245] (often lost during other strategies to combat obesity, such as a very low-calorie diet [VLCD] [246]). One potential mechanism for this is via enhancement of MPS, as in older men GLP-1 infusions have been shown to overcome anabolic resistance to nutrition [247].

Finally, new compounds are currently undergoing clinical trials to determine their effects on muscle mass and functional decline with ageing. For example, Bimagrumab, a human monoclonal antibody which binds to the activin type II receptors on skeletal muscle prevents the binding of muscle growth inhibitors such as myostatin [248]. To date, Bimagrumab has been demonstrated to improve both SKM mass and function in early clinical trials of older adults with sarcopenia [249]. It has also, in adults (∼60 years of age) with Type 2 diabetes and obesity, been shown (alongside diet and exercise counselling) to elicity greater decreases in total fat mass and glycated hemoglobin (HbA1c) and larger increases in SKM mass than those in a placebo comparator group.

## Review summary

The available evidence strongly indicates that the loss of muscle mass and function associated with advancing age is largely (but not exclusively, i.e., regenerative capacity and SKM vascularisation each also have a likely role to play [250]) due to declines in MPS and alterations to motor pathways. However, the relative contribution of each is not well established and the two have not been explored in tandem within individual studies. Beyond mechanistic insight, the relative contribution of each may be of limited importance as established exercise interventions (particularly RET) have been shown to benefit both factors. Alongside nutritional (e.g., protein) interventions, this provides a collective stimulus for increasing MPS and therefore can go some way to ameliorate losses of SKM mass and strength. Additionally, there is future promise with the recent developments and current investigation of repurposed pharmaceutical agents such as rapamycin and Bimagrumab, offering potential alternatives particularly for groups unable to exercise and/or get appropriate nutrition such as those in intensive care. Beyond this, enhanced mechanistic insight into the precise processes responsible for SKM dysfunction with advancing age may facilitate rational design of novel therapeutics for what is clearly a topic issue of ever-growing importance.

## Data Availability

As a review article, a data availability statement is not relevant.

## Open Access

Open access for this article was enabled by the participation of University of Nottingham in an all-inclusive *Read & Publish* agreement with Portland Press and the Biochemical Society under a transformative agreement with JISC.

## Abbreviations

AA: amino acid
CRF: cardiorespiratory fitness
EAA: essential AA
GLP-1: glucagon like peptide-1
HMB: β-hydroxy-β-methylbutyrate
NMES: neuromuscular electrical stimulation
PIC: persistent inward currents
RDA: recommended daily allowance
SKM: skeletal muscle
